# Effects of Reducing the South and Reinforcing the North Method on Inflammatory Injury Induced by Hyperlipidemia

**DOI:** 10.1155/2021/1860508

**Published:** 2021-09-20

**Authors:** Hongjin Wu, Weiwei Dai, Libo Wang, Jie Zhang, Chenglong Wang

**Affiliations:** Central Laboratory for Science and Technology, Longhua Hospital Shanghai University of Traditional Chinese Medicine, Shanghai 200032, China

## Abstract

Inflammation is the pathophysiological basis of hyperlipidemia-related disease (HRD). Reducing the south and reinforcing the north method (RSRN) has a positive effect on HRD. However, the pharmacological mechanisms of RSRN are still unclear in the treatment of HRD. We obtained RSRN compounds from the Traditional Chinese Medicine Systems Pharmacology (TCMSP) and identified potential targets of these compounds through target fishing based on the TCMSP databases. Next, we identified the HRD targets by using multiple databases. Then, the overlapping genes between the RSRN potential targets and the HRD targets were used to establish a protein-protein interaction (PPI) network, and we further analyzed their interactions and identified the major hub genes in this network. Subsequently, the Metascape database was utilized to conduct the enrichment of Gene Ontology biological processes (GO) and Kyoto Encyclopedia of Genes and Genomes (KEGG) pathways. A total of 187 potential active components and 106 related core targets were obtained and identified overall. Then after the Metascape enrichment analysis, a total of 148 KEGG pathways were screened, which were mainly associated with AGE-RAGE signaling pathway, PI3K-Akt signaling pathway, TNF signaling pathway, and NF-kappa B signaling pathway. Furthermore, 34 hub genes, such as AKT1, NF-*κ*Bp65(RELA), I*κ*B*α*(CHUK), MAPK8, and MAPK14, CCND1, were considered potential therapeutic targets. Furthermore, evaluations of protein levels of NF-*κ*Bp65, I*κ*B*α*, TNF-*α*, IL-1 *ß*, and IL-6 were performed for experimental validation. RSRN can reduce the expression of NF-*κ*Bp65 protein, increase the level of I*κ*B*α* protein, and reduce the protein levels of TNF-*α*, IL-1*β*, and IL-6 in ovariectomized rats. The results indicate that the mechanism of RSRN against inflammation may be related to AKT1, NF-*κ*Bp65, I*κ*B*α*, MAPK8, and MAPK14, as well as TNF, NF-kappa B, PI3K-Akt signaling pathways.

## 1. Introduction

The decreased ovarian function and estrogen levels in menopausal women contributes to the increase in the prevalence of dyslipidemia, osteoporosis, and urinary tract infection, of which hyperlipidemia is the most insidious [1]. Previous studies have reported that hyperlipidemia could lead to atherosclerosis (AS), coronary heart disease (CHD), and Alzheimer's disease [2, 3]. There should be necessary interventions to be done for preventing the hyperlipidemia related diseases (HRD), thus to reduce the incidence rate and mortality of AS and CHD in middle-aged and elderly women.

Traditional Chinese medicine (TCM) believes that the kidney is the congenital life basis. When women are in the menopausal period, kidney-Qi declines; Tiangui will be exhausted; Chong and Ren become deficient; essence and blood become insufficient; all these conditions can lead to imbalance between Yin and Yang and Zang-fu organs disorders, resulting in menopausal syndromes and hyperlipidemia-related diseases [4]. The pathogenesis of HRD belongs to the syndrome of deficiency of origin and excess of standard, while kidney Yin deficiency is the foundation, and heart fire is excessive, and essence deficiency and blood stasis is the standard [5]. In clinic, method of nourishing kidney and clearing heart can effectively alleviate and control these syndromes. Therefore, reducing the south (clearing heart) and reinforcing the north (nourishing kidney essence) may be the foundation of the HRD treatment. Reducing the south and reinforcing the north formula (RSRN) consist of eight herbs: *Epimedium sagittatum* (Siebold &Zucc.) Maxim. (ES); *Curculigo orchioides* Gaerth. (CO); *Angelica sinensis* (Oliv.) Diels. (AS); *Phellodendron chinense* Schneid. (PC); *Anemarrhena asphodeloides* Bge. (AR); *Morinda officinalis* How (MO); *Radix Salvia* (RS); and *Coptidis Rhizoma* (CR). Pharmacological studies on RSRN have assessed its regulation of the sex hormone and lipid metabolism and its prevention of the atherosclerosis and cardiovascular disease during the climacteric period [6, 7]. It was reported [8, 9] that Modified RSRN could enhance the myocardial microvascular density, improve the endothelial secretion function and hemorheology, and regulate the endocrine system and lipid metabolism in ovariectomized rats.

**Figure 1:**
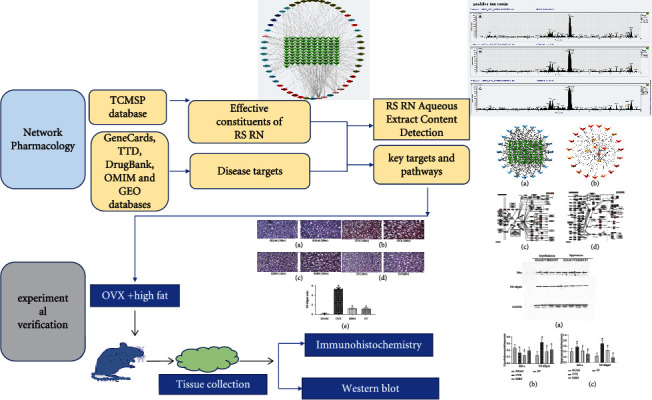
The detailed flowchart of the current study.

Network pharmacology is a kind of new method for investigating the pharmacological mechanisms of TCM based on system biology and multidirectional pharmacology [10]. Due to the complexity of the TCM components and the uncertainty of their targets, conventional pharmacology research methods are difficult to fully elucidate the potential molecular mechanism of TCM compound in the treatment of diseases. In this study, we dissected the mechanisms of RSRN in treating hyperlipidemia and atherosclerosis and identified compounds related to the RSRN with the help of network pharmacology method based on multiple databases; we obtained the compounds potential targets via target fishing and verified the results through experiment. The detailed procedures can be seen in Figure 1.

## 2. Materials and Methods

### 2.1. Identification of Main Active Components and Related Targets

Herbs were first confirmed by the comprehensive database of traditional Chinese medicine (TCMID), http://119.3.41.228 : 8000/TCMID/prescription search/). Then all of the constituent data of RSRN were obtained from the TCMSP database (http://lsp.nwu.edu.cn/tcmsp.php) [11]. The active components were further identified using the parameters of the oral bioavailability (OB); we set the threshold of OB at ≥30% and the drug likeness (DL) at ≥0.18 [12]. Then we used the TCMSP platform to predict the targets of active ingredients. The target information was obtained by correcting all the retrieved targets to their official names (official symbol) based on the UniprotKB search function in the protein database (UniProt) (http://www.uniprot.org/).

### 2.2. Disease Targets Identification by Multiple Databases

“Hyperlipidemia,” “atherosclerosis,” and “Alzheimer's disease” were selected as the key words for the retrieval of disease targets from the GeneCards database (https://www.genecards.org/), TTD database (Therapeutic Target Database, http://bidd.nus.edu.sg/group/cjttd/), DrugBank database (https://www.durgbank.ca/), OMIM database (Online Mendelian Inheritance in Man (https://www/omim.org/), and then the acquired disease targets were intersected by FunRich3.1.3 software. GSE57691 was screened and download from the GEO database (gene expression omnibus, https://www. ncbi. nlm. nih.gov/geo/) with “hyperlipidemia” and “atherosclerosis” as the key words, including 58 cases of disease group (49 cases of abdominal aortic aneurysm and 9 cases of aortic occlusive disease) and 10 cases of normal control group. We merged the above results and deleted the duplicate genes to obtain the final disease target genes by *R* 4.0.2 software.

### 2.3. Construction and Analysis of Drug-Disease Target Network

Based on previous steps, drug-disease crossover genes were screened with *R* software using the Venn Diagram package. The String 11.0 database (http://string-db.org/) was used to analyze the intersecting protein-protein interactions (PPIs) and then Cytoscape3.6.0 was used to determine the drug-disease target network. The “centiscape” plug-in was used to calculate the degree of freedom of drug-disease target. The higher the “degree” value, the greater the probability of playing the main function [13]. Using the Bisogenet and CytoNCA plug-ins of Cytoscape software (version 3.6.0), we set the parameters of degree centrality (DC > 61) and the topology intermediateness (BC > 600) and constructed the core target gene topology network; furthermore, we screened out the important candidate genes.

### 2.4. GO and KEGG Pathway Analysis

The screened drug-disease targets were evaluated by functional enrichment analyses. GO analysis was performed in three categories, namely biological processes (BP), cellular component (CC), and molecular function (MF), and the KEGG signaling pathway analysis was performed with *R* software using the Bioconductor package. The KEGG pathway was introduced into Cytoscape 3.6.0. According to the degree value, the enrichment degree of pathway and gene was displayed, and the network diagram was drawn.

### 2.5. Herbal Preparation

**Figure 2:**
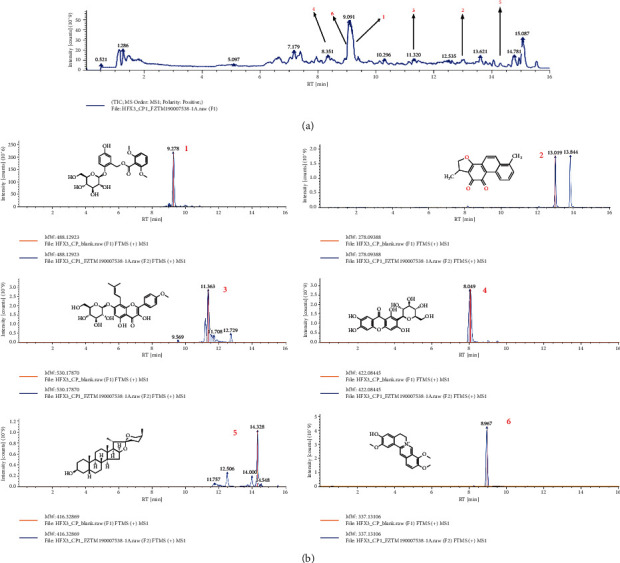
HPLC-MS mass specterometry analysis of the RSRN. (a) First-order spectrogram positive ion mode. (b) Second-order spectrogram positive ion mode. (c) Mass spectrometry of the main components; 1. Curculigoside; 2. Dihydrotanshinoen I; 3. Icarisid I; 4. Mangiferin; 5. Sarsasapogenin; 6. Jatrorrhizine.

We prepared RSRN using the following constituents: 30 g of *Curculigo orchioides* Gaertn (Xian Mao), 30 g of *Epimedii* Folium (Yin yang huo), 15 g of *Morindae officinalis* Radix (Ba ji tian), 15 g of *Angelicae Sinensis* Radix (Dang gui), 12 g of *Phellodendron chinense* Cortex (Huang bo), 10 g of *Anemarrhenae Rhizoma* (Zhi mu), 12 g of *Salviae Miltiorrhizae* Radix et Rhizoma (Dan shen), and 6 g of *Coptidis Rhizoma* (Huang lian). We obtained all herbs from Longhua Hospital Shanghai University of TCM, China. The herbs were mixed, soaked in water for 0.5 h, and decocted for 1 h in 5% v/w distilled H_2_O at 100°C. Subsequently, the filtrate was collected, and the residue decocted for another hour with 5% v/w distilled water. Next, the filtrate was concentrated (RE-3000B, Ya-rong Biochemical Instrument Shanghai Co., Ltd) and lyophilized (LGJ-10D, Four-ring Science Instrument Plant Beijing Co., Ltd), and the resulting RSRN powder was kept at −20°C until use. HPLC was used to determine the components in RSRN, and determination of curculigoside, Dihydrotanshinoen I, Icarisid I, Mangiferin, Sarsasapogenin, Jatrorrhizine in RSRN is shown in Figure 2.

### 2.6. Animals and Administration

We obtained 40 healthy eight-week-old female Sprague-Dawley (SD) rats (mean weight: 200 ± 20 g) from Shanghai Slack Laboratory Animals Co., Ltd. [license number: SCXK (Hu) 2012–0002]. All the rats were housed in an air-conditioned room with 12 h light-dark cycles at a constant temperature (22–26°C) and humidity (50% ± 10%). Further, the rats were provided with rodent chow and tap water ad libitum. The animal model of climacteric atherosclerosis was established by bilateral ovariectomy and high-fat diet [14]. After acclimation for one week, the rats underwent either ovariectomy (*n* = 30) or sham operation (*n* = 10) under anesthesia using an intraperitoneal injection of 30 mg/kg pentobarbital sodium. Nine days after surgery, we randomly divided 30 OVX rats into the following three groups: OVX (high-fat emulsion [15] with an equal volume of ddH_2_O; 1 mL/100 g/day), RSRN (high-fat emulsion with RSRN; 5.46 g/kg/day), and EV groups (high-fat emulsion with estrogen valerate; 0.1 mg/kg/day). The sham-operated rats (SHAM group) received a normal diet with an equal volume of ddH_2_O; 1 mL/100 g/day. During the experimental period, the rats underwent weekly weight measurements. All procedures were approved by the Department of Laboratory Animal Science Longhua Hospital Shanghai University of TCM. The animal welfare and experimental procedures were conducted in strict accordance with the guidelines for the care and use of experimental animals and the ethics of Shanghai University of TCM. For the next six weeks after the last administration, the rats were food- and water deprived for 12 h. The rats were anaesthetized by diethyl ether inhalation. The brain and the blood were taken for further use.

### 2.7. Immunohistochemistry Analysis

Immunohistrochemistry procedure was performed as previously reported protocol [16]. The sections were incubated with the primary antibody for NF-*κ*B p65 overnight at 4°C (#8242; 1 : 100, Cell Signalling Technology, MA,USA). After washing, the sections were incubated with the secondary antibody (#MR-R100; MR Biotech, Shanghai, China) for 1 h and then stained with 3,3′-diaminobenzidine (DAB) (Biyuntian Institute of Biotechnology, Jiangsu, China). Signals were visualized by light microscopic observation. The results were analyzed by using Image *J* software version1.50i (National Institute of Health, USA). The investigators used the software to measure the ratio of positive area (A%).

### 2.8. Western Blotting Analysis

The protein samples were separated by 10% SDS-PAGE transferred to PVDF membrane. The membrane was blocked with 1% BSA in TBST for 30 min, the primary antibodies including NF-*κ*Bp65 (#8242; 1 : 1000; Cell Signalling Technology, MA, USA), I*κ*B *α* (#4812; 1 : 1000; Cell Signalling Technology, MA, USA), TNF-*α* (#sc-52746; 1 : 1000; Santa Curz Biptechnology, USA), IL-1*β* (#sc-52012; 1 : 1000; Santa Curz Biptechnology, USA), IL-6 (#sc-32296; 1 : 1000; Santa Curz Biptechnology, USA) were incubated at 4°C overnight (≥12 h), and secondary antibodies for GAPDH (#5174T; 1 : 5000; Cell Signalling Technology, MA, USA) and m-IgGk BP-HRP (#sc-516101; 1 : 5000; Santa Curz Biptechnology, USA) were used as the internal reference antibodies. Washed with TBST 5 min × 3 times, chemiluminescence imaging was performed with ECL luminescent liquid.

### 2.9. Statistical Analyses

Using SPSS20.0 statistical analysis software, the data were expressed as means ± standard deviation (SD). The significant difference was expressed as *p* < 0.05. One-way ANOVA was used to compare the experimental results among groups. LSD test (meeting the requirement of homogeneity of variance) or Dunnett's T3 (not meeting the requirement of homogeneity of variance) was used for further pairwise comparison.

## 3. Results

### 3.1. Active Components of RSRN

A total of 187 active components were identified from the TCMSP databases by the ADME thresholds (OB ≥ 30%, DL ≥ 0.18), including 8 components of *Curculigo orchioides* Gaertn (Xian Mao), 22 components of *Epimedii* Folium (Yin yang huo), 21 components of *Morindae officinalis* Radix (Ba ji tian), 2 components of *Angelicae Sinensis* Radix (Dang gui), 38 components of *Phellodendron chinense* Cortex (Huang bo), 17 components of *Anemarrhenae Rhizoma* (Zhi mu), 65 components of *Salviae Miltiorrhizae* Radix et Rhizoma (Dan shen), and 14 components of *Coptidis Rhizoma* (Huang lian) (Table 1).

### 3.2. Analysis of “RSRN-Compound-Target” Network

**Figure 3:**
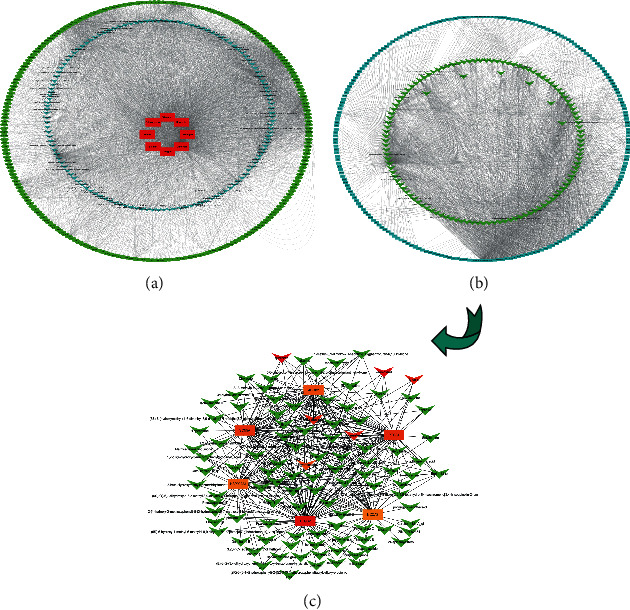
The “RSRN-compound-target” network and the key targets: (a) the RSRN-active component-target network, the red rectangle represents the name of drug, the blue inverted triangle represents the components, the green circle represents the targets; (b) the active component-key target network, the blue rectangle represents the key targets, the green inverted triangle represents the components.

We conducted target fishing for these 187 active ingredients using the TCMSP databases based on chemical similarity and obtained 225 related targets. We evaluated the relationships between the components and targets with a constructed “RSRN-compound-target” network, which had a total of 365 nodes and 4841 edges (Figure 3(a)). The inverted triangles represent the active compounds, and the circles represent their targets. The network topology was analyzed by using centiscape plug-in, and the degree value of the topological network was 26.53, the betweenness value was 469.26, the closeness value was 0.0012, and the eigenvector value was 0.039. The top 10 compounds and top 6 key target of RSRN are shown in Table 2. The top six targets were PTGS2 (degree = 231), PTGS1 (degree = 148), SCN5A (degree = 137), HSP90AA1 (degree = 131), NCOA2 (degree = 130), and ADRB2 (degree = 127) (Figure 3(b) and 3(c)).

### 3.3. Disease Targets Acquisition and Analysis

**Figure 4:**
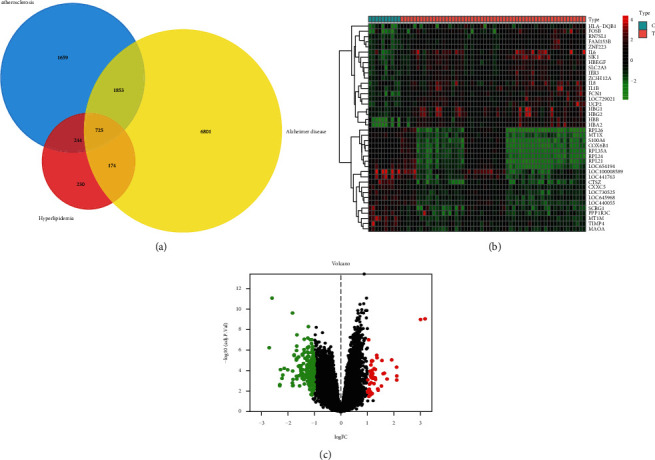
Disease targets analysis: (a) “hyperlipidemia,” “atherosclerosis,” and “Alzheimer's disease” were selected as the key words for the retrieval of disease targets; (b, c) the cluster diagram and volcano map of the differentially expressed genes in GSE57691 data set, red represents upregulated and green represents downregulated.

There were 1373 disease targets related to hyperlipidemia, 4481 targets related to atherosclerosis, and 9553 targets related to “hyperlipidemia,” which were selected from GeneCards, TTD, DrugBank, and OMIM database. The above disease targets were analyzed by Venn map (Figure 4(a)). The data set GSE57691 was selected from the GEO database for screening differentially expressed genes. Then differential expression analysis on the data was performed using the limma software package. Compared with control samples, a total of 269 genes were significantly differentially expressed in hyperlipidemia samples, 47 were upregulated, and 221 were downregulated. The differentially expressed genes are shown in the cluster diagram (Figure 4(b)) and volcano map (Figure 4(c)). A total of 974 disease targets were obtained by merging above disease targets in the end.

### 3.4. Construction of Drug-Disease Target Network

**Figure 5:**
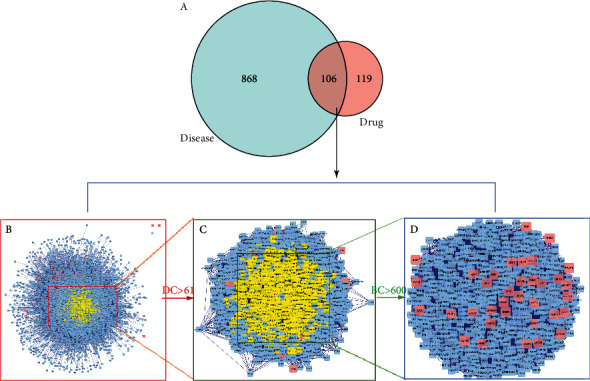
The drug-disease target network. (a) The Venn diagram for drug and disease targets. The overlap targets mean the potential therapeutic gene for RSRN when treating hyperlipidemia-related disease; (b) there were 6664 nodes and 146555 edges in the network; (c) the first screening threshold was DC >61, which resulted in 1350 nodes and 58513 edges; (d) the second screening threshold was BC >600, and 609 nodes and 25745 edges remained.

After the construction of the Venn diagram, 106 targets between the 974 disease targets and 225 related targets of RSRN were selected as the potential targets in the treatment of hyperlipidemia-related diseases (Figure 5(a)). The protein interaction relationship was obtained by using BisoGenet plug-in of Cytoscape software (Figure 5(b)). Using the CytoNCA plug-in of Cytoscape software, the core disease-drug targets was analyzed and confirmed. The selection criteria were set as follows: DC value > 61 (Figure 5(c)) (yellow part in the figure was qualified), and BC value > 600 (Figure 5(d)). The red rectangle in the topological network represents the last selected target genes, including AKT1, AR, NF-*κ*B, CASP3, mTOR, ERBB2, CHUK, CAV1, MAPK8, MAPK14, HIF1A, PPARG, RELA, NR3C1, ESR2, FOS, CDK4, GSK3B, HSPB1, MYC, MDM2, EGFR, HSP90AA1, HSPA5, NOS2, ADRB2, VCAM1, APP, ESR1, XIAP, CASP8, Bax, ICAM1, SOD1.

### 3.5. Functional Enrichment Analysis

**Figure 6:**
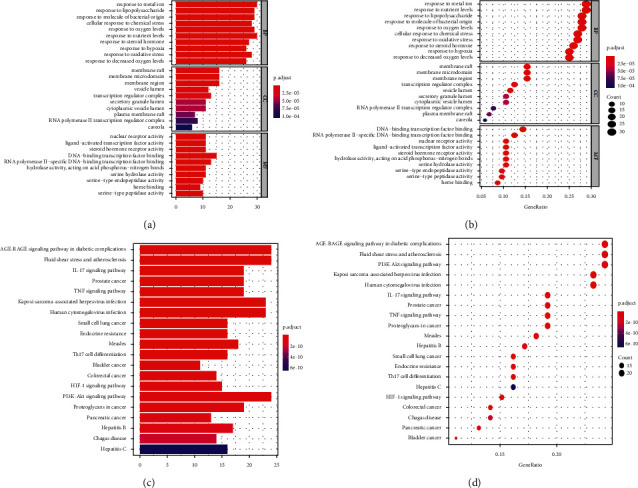
Functional enrichment analysis. (a, b) GO functional enrichment analysis; (c, d) KEGG functional enrichment analysis.

**Figure 7:**
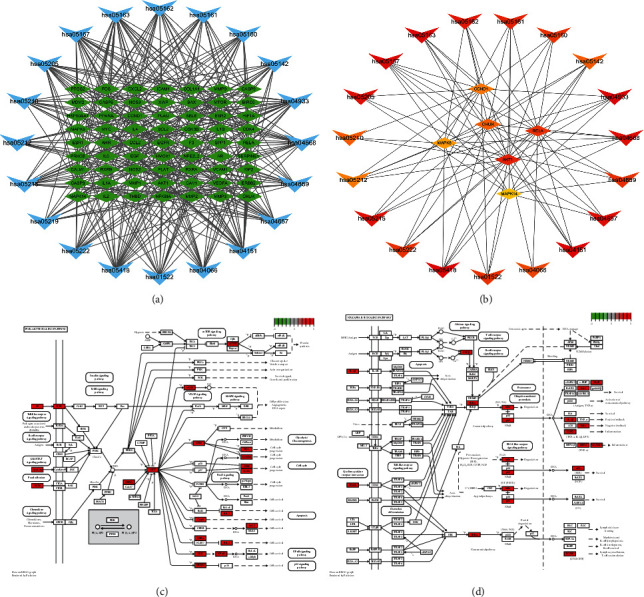
KEGG network and key pathways: (a) KEGG pathways target genes network, blue inverted triangle represents KEGG pathways, green rectangle represents KEGG pathway-related genes; (b) top 25 pathways and target genes; (c) PI3K-AKT signal pathway; (d) NF-KAPPA B signal pathway.

The 106 potential targets were then subjected to GO and KEGG analysis to explore the links between the functional units, their potential significance in the biological systems network. The GO terms were determined in the following categories (Figures 6(a) and 6(b)): 1900 biological processes (BP), 43 cellular components (CC), and 140 molecular functions (MF) branches. In the category BP, the genes were associated with response to metal ion (GO:0010038), response to nutrient levels (GO:0031667), response to lipopolysaccharide (GO:0032496), and response to molecular of bacterial origin (GO:0002237). In the category CC, the genes were associated with cell components such as membrane raft (GO:0045121), membrane micro domain (GO:0098857), membrane region (GO:0098589), and transcription regulator complex (GO:0005667). In the category MF, the genes were associated with DNA binding transcription factor binding (GO:0140297) and RNA polymerase II specific DNA binding transcription factor binding (GO:0061629). The KEGG enrichment result indicated that the genes were associated with AGE-RAGE signaling pathway (hsa04933), fluid shear stress and atherosclerosis (hsa05418), PI3K-Akt signaling pathway (hsa04151), TNF signaling pathway (hsa04668), and NF-kappa B signaling pathway (hsa04064) (Figures 6(c) and 6(d)). The KEGG network included 83 nodes and 360 edges (Figure 7(a)). The top six target genes were AKT1 (protein-serine-threonine kinase 1) (degree = 17), RELA (nuclear factor kappa B p65, NF-*κ*B p65) (degree = 16), CHUK (conserved helix-loop-helix ubiquitous kinase, also known as I*κ*B kinase *α*, IKK*α*, or IKK1) (degree = 14), CCND1 (Cyclin D1) (degree = 13), MAPK8 (mitogen-activated protein kinase 8) (degree = 12), and MAPK14 (mitogen-activated protein kinase 14) (degree = 11) (Figure 7(b)). The PI3K-Akt signaling pathway and NF-kappa B signaling pathway were performed by *R* software (Figures 7(c) and 7(d)), and the red mark represents the potential target of RSRN intervention.

### 3.6. RSRN Downregulated the Expression of NF-*κ*Bp65 Protein in Hypothalamus of Ovariectomized Rats

**Figure 8:**
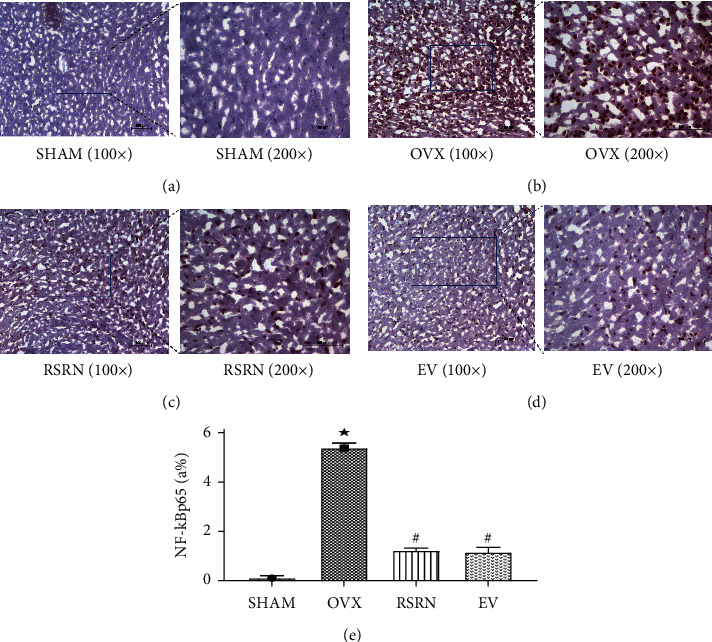
The expression of NF-*κ*Bp65 in hypothalamus. (a–d) The expressions of NF-kBp65 in hypothalamus tissue were analyzed by immunohistochemistry (×200). (e) Values are presented as the mean ± standard deviation (SD), *n* = 3 per group. ^★^*p* < 0.05, compared with the SHAM group; ^#^*p* < 0.05, compared with the OVX group.

The *immunohistochemistry* results showed that the positive expression of NF-*κ*Bp65 cells included glial cells, which were located in cytoplasm or nucleus (Figure 8(a)–8(e)). Compared with the SHAM group, the expression of NF-*κ*Bp65 protein in hypothalamus in OVX group was significantly increased (*p* < 0.05), with a large number of positive expression cells and dark brown color. Compared with the OVX group, the expression of NF-*κ*Bp65 protein in hypothalamus in RSRN group and EV group was significantly decreased (*p* < 0.05).

### 3.7. RSRN Regulated the Expression of NF-*κ*Bp65, I*κ*B*α*, TNF*α*, IL-1*β*, and IL-6 in Brain of Ovariectomized Rats

**Figure 9:**
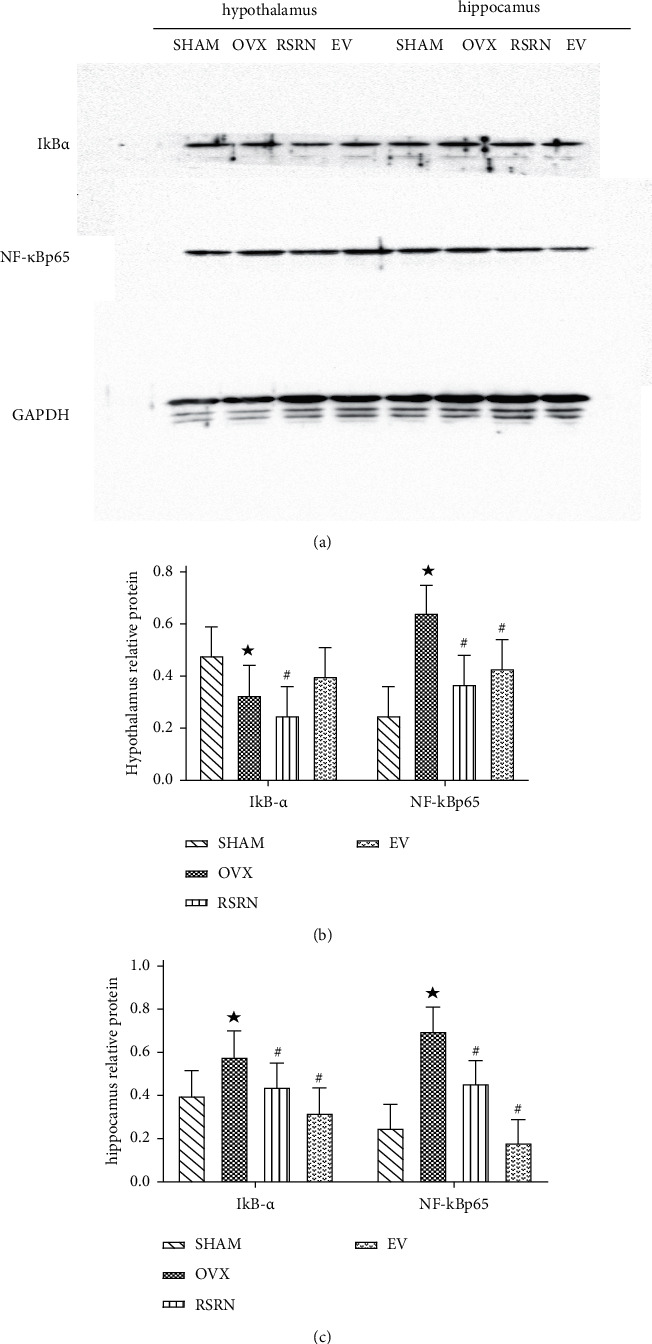
Protein expression of I*κ*B*α* and NF-*κ*Bp65 by western blot: (a) gene levels of NF-*κ*Bp65, I*κ*B*α* in hypothalamus and hippocampus by western blot; (b) semi-quantitative analysis of NF-*κ*Bp65, I*κ*B*α* proteins expression compared with GAPDH. Values are presented as the means ± standard deviation (SD), *n* = 3 per group. ^★^*p* < 0.05, compared with the SHAM group; ^#^*p* < 0.05, compared with the OVX group.

**Figure 10:**
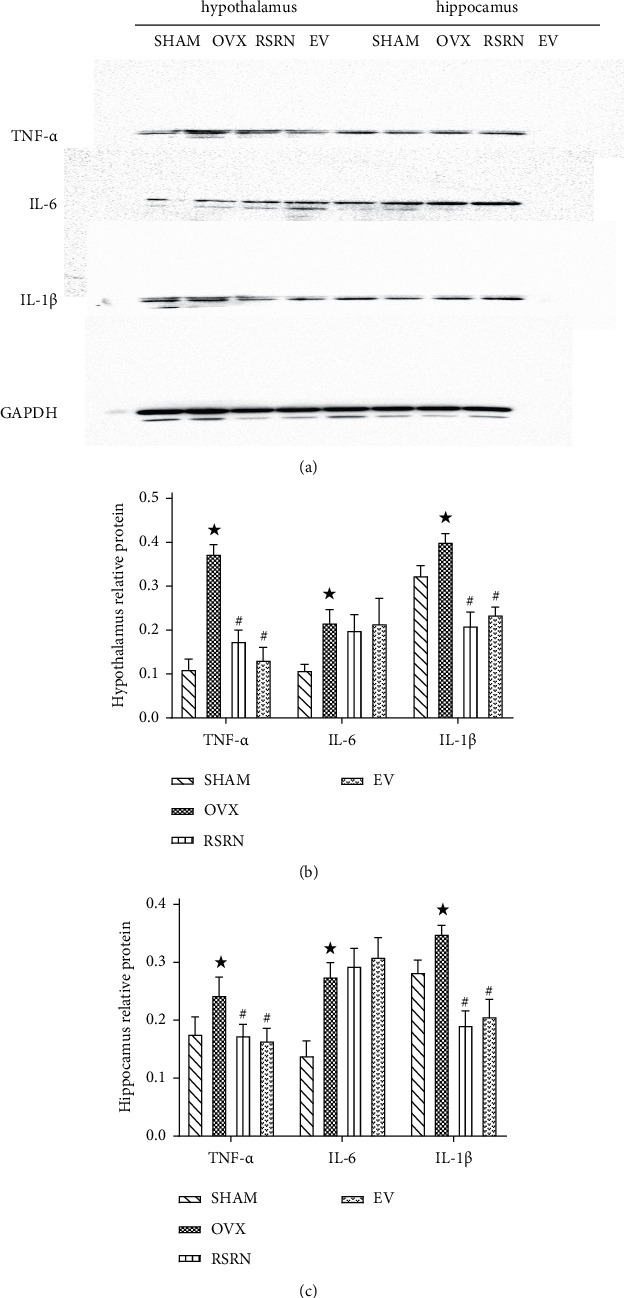
Protein expression of TNF*α*, IL-1*β*, and IL-6 by western blot: (a) gene levels of TNF*α*, IL-1*β*, and IL-6 in hypothalamus and hippocampus by western blot; (b) semi-quantitative analysis of TNF*α*, IL-1*β*, and IL-6 proteins expression compared with GAPDH. Values are presented as the means ± standard deviation (SD), *n* = 3 per group. ^★^*p* < 0.05, compared with the SHAM group; ^#^*p* < 0.05, compared with the OVX group.

Western blotting results showed that the expression levels of NF-*κ*Bp65, TNF*α*, IL-1*β*, and IL-6 in OVX group were significantly higher than those of the SHAM group (*p* < 0.05), while the expression of I*κ*B*α* was significantly decreased (*p* < 0.05). Compared with the OVX group, the protein expression levels of NF-*κ*Bp65, TNF*α*, IL-1*β*, and IL-6 in RSRN group were significantly decreased (*p* < 0.05), while the expression of I*κ*B*α* protein was significantly increased (*p* < 0.05) (Figure 9(a)–9(c); Figure 10(a)–10(c)).

## 4. Discussion

The method of reducing the south and reinforcing the north (RSRN) can nourish kidney essence and purging the heart. It is also called the method of purging fire and replenishing water. The method of RSRN in this study was used to coordinate yin and yang and to prevent postmenopausal-related diseases. There were 187 active components in RSRN, of which quercetin, kaempferol, Stigmasterol, luteolin, beta-sitosterol, and Anhydroicaritin were the main active components. Kaempferol and quercetin may have hypoglycemic, lipid-lowering, anti-inflammatory, antioxidant, and anticancer effects [17]. It was reported that Kaempferol can increase lipid metabolism by increasing PPAR*α* level, decreasing SREBPs level, and promoting expression of ACO and CYP4A1, so as to reduce visceral fat accumulation and improve hyperlipidemia in obese rats fed with high-fat diet [18]. Quercetin can improve cholesterol reverse transport by upregulating the expression of ABCA1 and ABCG1 protein and enhancing the cholesterol acceptance of HDL and ApoA1 by reducing oxidation so as to reduce lipid accumulation [19]. Research [20] showed that luteolin could reduce the activation of PI3K/Akt induced by EGF and reduce the phosphorylation of EGFR, Akt, p38, and extracellular signal regulated kinase (ERK). Studies [21] found that pretreatment with luteolin could reduce the production of proinflammatory cytokines such as TNF-*α*, IL-6, and inflammatory mediator nitric oxide (NO) which produced by lipopolysaccharide (LPS)-stimulated MH-S cells of mouse alveolar macrophages. Studies have illustrated that *ß*-sitosterol exerts cholesterol-lowering, antioxidant, and anti-inflammatory effects [22, 23]. The above studies showed that the main active ingredients of RSRN had anti-inflammatory, antioxidation stress, hypoglycemic and lipid-lowering effects, and cardiovascular system protection.

A total of 106 drug-disease core targets were obtained, and the genes were used to constructed the core target gene topology network and the KEGG network. The top six target genes from the KEGG network were merged with the candidate genes from the core target gene topology network; we obtained the key target genes including AKT1, RELA, CHUK, MAPK8, MAPK14, and CCND1. The key targets are mainly involved in NF-*κ*B/MAPK signaling pathway, PI3K-Akt signaling pathway, atherosclerosis, and inflammation-related signaling pathways. Akt is the central link of PI3K/Akt signaling pathway, and it plays an important role in regulating cell survival, protein synthesis, angiogenesis, and insulin-dependent metabolic cell response [24]. Akt can inhibit apoptosis, phosphorylate caspase-9 precursor [25, 26]. Nuclear factor *κ*B (NF-*κ*B) plays an important role in the regulation of gene transcription related to inflammation, cell proliferation, differentiation and apoptosis, immune response, and tumor formation; the human NF-*κ*B family consists of five members: P50/P105, p52/P100, p65/RELA, RELB, and c-Rel, which are encoded by NF-*κ*B1, NF-*κ*B2, RELA, RELB, and REL genes [27]. CHUK (also known as IKK *α*, IKK *α*) is the upstream component of signal transduction pathway that directly enters the nucleus to regulate gene expression, and it is also a component of activating cytokine protein complex, studies have shown that gene mutation of CHUK is associated with hypertension and lipid abnormality [28–30]. These studies indicated that the key targets have the functions of regulating lipid metabolism, glucose metabolism, and immune regulation, which were of great significance for the prevention and treatment of hyperlipidemia, and also had certain effects on the complications of atherosclerosis and Alzheimer's disease.

The core targets of RSRN-active ingredients in the treatment of hyperlipidemia-related diseases may involve in the response to lipopolysaccharide, oxidative stress, and DNA binding transcription factor binding. Oxidative stress plays an important role in the pathogenesis of atherosclerosis (AS), hypertension, metabolic syndrome, hypercholesterolemia, Alzheimer's disease, aging, and cancer [31]. Lipopolysaccharide (LPS) activates downstream target genes by activating TLR4/NF-*κ*B signaling pathways, thus to release inflammatory factors such as TNF-*α*, IL-8, IL-1, and IL-6 [32]. Endotoxin is an inflammatory reaction promoter of LPS in the outer membrane of Gram-negative bacteria; it is the main ligand of Toll like receptor; and it has been confirmed that endotoxin plays an important role in the process and progress of AS [33, 34].

The main pathways of RSRN may be involved in AGE-RAGE signaling pathway, cell fluid shear stress and atherosclerosis, and PI3K-Akt signaling pathway. Advanced glycosylated compounds (AGEs) are complex compounds produced by nonenzymatic glycosylation and oxidation of proteins, lipids, and nucleic acids; they can activate AGE-RAGE signaling pathway, MAPK signaling pathway, and NF-*κ*B signaling pathway, leading to the expression of proinflammatory cytokines such as IL-1, IL-6, and TNF–*α*, and the release of VCAM1, VEGF, and RAGE, so as to promote the development of atherosclerosis [35]. Cell fluid shear stress and atherosclerotic pathway play an important role in the development of dyslipidemia to atherosclerosis; it has been reported that the wall concentration of lipid in the slow flow area was higher than that in the high-speed laminar flow area, and the action time of lipid and arterial wall was prolonged, which was easy to cause atherosclerosis, and it also can promote oxidative stress, increase the production of ox LDL, upregulate the expression of NF-*κ*B, thus to promote inflammatory response [36, 37]. PI3K/Akt signaling pathway plays an important role in lipid metabolism and inflammation regulation; inhibition of PI3K/Akt signaling pathway can significantly reduce serum-free fatty acids, cholesterol, and triglyceride [38, 39], and it also inhibited the secretion of proinflammatory mediators such as TNF-*α* and IL-1 *ß* [40]. It can be speculated that RSRN may regulate dyslipidemia and prevent or delay the occurrence and development of AS through regulation of endocrine, metabolic, and inflammatory pathways.

Studies have found that hyperlipidemia is closely related to hypothalamic inflammatory response. Hypothalamic inflammation leads to the occurrence of obesity-based metabolic diseases. A short-term high-fat diet can increase the expression of biomarkers and promote inflammatory response in the basal hypothalamus to form a transient inflammation. Under the condition of long-term high-fat diet, hypothalamic glial hyperplasia, and nerve injury can promote the occurrence of hypothalamic inflammation [41]. In this experimental study, the results showed that the expression of NF-*κ*B p65, TNF-*α*, IL-1*β*, IL-6 in hypothalamic nucleus of OVX group was significantly increased, which indicated that NF-*κ*B signal pathway and inflammatory cytokines were activated under the stimulation of intracellular and extracellular signals; after treatment with RSRN, the expression of activated NF-*κ* B (p65) in nucleus was significantly reduced, and the expression of TNF-*α*, IL-1*β*, and IL-6 was also significantly reduced in RSRN group; the results showed that RSRN could inhibit the activity of NF-*κ*B and reduce the release of inflammatory cytokines. In the future, it is necessary to further explore the relationship between target protein and the upstream and downstream molecules of the signaling pathway and the specific regulatory mechanisms and confirm the curative effect through clinical trials.

## 5. Conclusion

In this study, a total of 187 potential active components and 106 related core targets were obtained and identified overall. Then after the Metascape enrichment analysis, RSRN may regulate AKT1, NF-*κ*Bp65, IKK *α*, TNF-*α*, IL-1 *β*, IL-6 through TNF signaling pathway, PI3K-Akt signaling pathway, and NF-kappa B signaling pathway, so as to regulate lipid metabolism, inflammatory response, and prevent or delay the development of atherosclerotic diseases. This study suggests that RSRN may be used in the treatment of hyperlipidemia and related diseases. Due to the limitation of database data and corresponding analysis algorithms, the results may be biased, and further in vitro and in vivo studies are needed to verify the results.

## Figures and Tables

**Table 1:** Detailed information on 106 active compounds from RSRN.

Mol ID	Components	OB%	DL	Herbs
MOL001506	Supraene	33.6	0.42	Ba ji tian
MOL002879	Diop	43.6	0.39	Ba ji tian
MOL002883	Ethyl oleate (NF)	32.4	0.19	Ba ji tian
MOL000358	Beta-sitosterol	36.9	0.75	Ba ji tian
MOL000359	Sitosterol	36.9	0.75	Ba ji tian
MOL006147	Alizarin-2-methylether	32.8	0.21	Ba ji tian
MOL009495	2-Hydroxy-1,5-dimethoxy-6-(methoxymethyl)-9,10-anthraquinone	95.9	0.37	Ba ji tian
MOL009496	1,5,7-Trihydroxy-6-methoxy-2-methoxymethylanthracenequinone	80.4	0.38	Ba ji tian
MOL009500	1,6-Dihydroxy-5-methoxy-2-(methoxymethyl)-9,10-anthraquinone	105	0.34	Ba ji tian
MOL009503	1-Hydroxy-3-methoxy-9,10-anthraquinone	104	0.21	Ba ji tian
MOL009504	1-Hydroxy-6-hydroxymethylanthracenequinone	81.8	0.21	Ba ji tian
MOL009513	2-Hydroxy-1,8-dimethoxy-7-methoxymethylanthracenequinone	112	0.37	Ba ji tian
MOL009519	(2R,3S) -(+)-3′,5-dihydroxy-4,7-dimethoxydihydroflavonol	77.2	0.33	Ba ji tian
MOL009524	3beta,20(R),5-alkenyl-stigmastol	36.9	0.75	Ba ji tian
MOL009525	3beta-24S(R)-butyl-5-alkenyl-cholestol	35.4	0.82	Ba ji tian
MOL009537	Americanin A	46.7	0.35	Ba ji tian
MOL009541	Asperuloside tetraacetate	45.5	0.82	Ba ji tian
MOL009551	Isoprincepin	49.1	0.77	Ba ji tian
MOL009558	2-Hydroxyethyl 5-hydroxy-2-(2-hydroxybenzoyl)-4-(hydroxymethyl)benzoate	62.3	0.26	Ba ji tian
MOL009562	Ohioensin-A	38.1	0.76	Ba ji tian
MOL000358	Beta-sitosterol	36.9	0.75	Dang gui
MOL000449	Stigmasterol	43.8	0.76	Dang gui
MOL001454	Berberine	36.9	0.78	Huang bo
MOL001458	Coptisine	30.7	0.86	Huang bo
MOL002636	Kihadalactone A	34.2	0.82	Huang bo
MOL013352	Obacunone	43.3	0.77	Huang bo
MOL002641	Phellavin_qt	35.9	0.44	Huang bo
MOL002643	Delta 7-stigmastenol	37.4	0.75	Huang bo
MOL002644	Phellopterin	40.2	0.28	Huang bo
MOL002651	Dehydrotanshinone II A	43.8	0.4	Huang bo
MOL002652	delta7-dehydrosophoramine	54.5	0.25	Huang bo
MOL002656	Dihydroniloticin	36.4	0.81	Huang bo
MOL002659	Kihadanin A	31.6	0.7	Huang bo
MOL002660	Niloticin	41.4	0.82	Huang bo
MOL002662	Rutaecarpine	40.3	0.6	Huang bo
MOL002663	Skimmianin	40.1	0.2	Huang bo
MOL002666	Chelerythrine	34.2	0.78	Huang bo
MOL000449	Stigmasterol	43.8	0.76	Huang bo
MOL002668	Worenine	45.8	0.87	Huang bo
MOL002670	Cavidine	35.6	0.81	Huang bo
MOL002671	Candletoxin A	31.8	0.69	Huang bo
MOL002672	Hericenone H	39	0.63	Huang bo
MOL002673	Hispidone	36.2	0.83	Huang bo
MOL000358	Beta-sitosterol	36.9	0.75	Huang bo
MOL000622	Magnograndiolide	63.7	0.19	Huang bo
MOL000762	Palmidin A	35.4	0.65	Huang bo
MOL000785	Palmatine	64.6	0.65	Huang bo
MOL000787	Fumarine	59.3	0.83	Huang bo
MOL000790	Isocorypalmine	35.8	0.59	Huang bo
MOL000098	Quercetin	46.4	0.28	Huang bo
MOL001131	Phellamurin_qt	56.6	0.39	Huang bo
MOL001455	(S)-canadine	53.8	0.77	Huang bo
MOL001771	Poriferast-5-en-3beta-ol	36.9	0.75	Huang bo
MOL001601	1,2,5,6-Tetrahydrotanshinone	38.75	0.36	Dan shen
MOL001659	Poriferasterol	43.83	0.76	Dan shen
MOL001771	Poriferast-5-en-3beta-ol	36.91	0.75	Dan shen
MOL001942	Isoimperatorin	45.46	0.23	Dan shen
MOL002222	Sugiol	36.11	0.28	Dan shen
MOL002651	Dehydrotanshinone II A	43.76	0.4	Dan shen
MOL002776	Baicalin	40.12	0.75	Dan shen
MOL000569	Digallate	61.85	0.26	Dan shen
MOL000006	Luteolin	36.16	0.25	Dan shen
MOL006824	*α*-Amyrin	39.51	0.76	Dan shen
MOL007036	5,6-Dihydroxy-7-isopropyl-1,1-dimethyl-2,3-dihydrophenanthren-4-one	33.77	0.29	Dan shen
MOL007041	2-Isopropyl-8-methylphenanthrene-3,4-dione	40.86	0.23	Dan shen
MOL007045	3*α*-Hydroxytanshinone?a	44.93	0.44	Dan shen
MOL007048	(E)-3-[2-(3,4-dihydroxyphenyl)-7-hydroxy-benzofuran-4-yl]acrylic acid	48.24	0.31	Dan shen
MOL007049	4-Methylenemiltirone	34.35	0.23	Dan shen
MOL007050	2-(4-Hydroxy-3-methoxyphenyl)-5-(3-hydroxypropyl)-7-methoxy-3-benzofurancarboxaldehyde	62.78	0.4	Dan shen
MOL007051	6-o-Syringyl-8-o-acetyl shanzhiside methyl ester	46.69	0.71	Dan shen
MOL007058	Formyltanshinone	73.44	0.42	Dan shen
MOL007059	3-Beta-hydroxymethyllenetanshiquinone	32.16	0.41	Dan shen
MOL007061	Methylenetanshinquinone	37.07	0.36	Dan shen
MOL007063	Przewalskin a	37.11	0.65	Dan shen
MOL007064	Przewalskin b	110.32	0.44	Dan shen
MOL007068	Przewaquinone B	62.24	0.41	Dan shen
MOL007069	Przewaquinone c	55.74	0.4	Dan shen
MOL007070	(6S,7 R)-6,7-dihydroxy-1,6-dimethyl-8,9-dihydro-7h-naphtho[8,7-g] benzofuran-10,11-dione	41.31	0.45	Dan shen
MOL007071	Przewaquinone f	40.31	0.46	Dan shen
MOL007077	Sclareol	43.67	0.21	Dan shen
MOL007079	Tanshinaldehyde	52.47	0.45	Dan shen
MOL007081	Danshenol B	57.95	0.56	Dan shen
MOL007082	Danshenol A	56.97	0.52	Dan shen
MOL007085	Salvilenone	30.38	0.38	Dan shen
MOL007088	Cryptotanshinone	52.34	0.4	Dan shen
MOL007093	Dan-shexinkum d	38.88	0.55	Dan shen
MOL007094	Danshenspiroketallactone	50.43	0.31	Dan shen
MOL007098	Deoxyneocryptotanshinone	49.4	0.29	Dan shen
MOL007100	Dihydrotanshinlactone	38.68	0.32	Dan shen
MOL007101	Dihydrotanshinone I	45.04	0.36	Dan shen
MOL007105	Epidanshenspiroketallactone	68.27	0.31	Dan shen
MOL002331	N-Methylflindersine	32.4	0.18	Huang bo
MOL002894	Berberrubine	35.7	0.73	Huang bo
MOL005438	Campesterol	37.6	0.71	Huang bo
MOL006392	Dihydroniloticin	36.4	0.82	Huang bo
MOL006401	Melianone	40.5	0.78	Huang bo
MOL006413	Phellochin	35.4	0.82	Huang bo
MOL006422	Thalifendine	44.4	0.73	Huang bo
MOL001607	ZINC03982454	36.9	0.76	Xian Mao
MOL003578	Cycloartenol	38.7	0.78	Xian Mao
MOL000358	Beta-sitosterol	36.9	0.75	Xian Mao
MOL004114	3,2′,4′,6′-tetrahydroxy-4,3′-dimethoxy chalcone	52.7	0.28	Xian Mao
MOL004125	Curculigoside B_qt	83.4	0.19	Xian Mao
MOL004146	Curculigosaponin C	39.3	0.19	Xian Mao
MOL000449	Stigmasterol	43.8	0.76	Xian Mao
MOL001510	24-Epicampesterol	37.6	0.71	Xian Mao
MOL001645	Linoleyl acetate	42.1	0.2	Yin yang huo
MOL001771	Poriferast-5-en-3beta-ol	36.9	0.75	Yin yang huo
MOL001792	DFV	32.8	0.18	Yin yang huo
MOL003044	Chryseriol	35.9	0.27	Yin yang huo
MOL003542	8-Isopentenyl-kaempferol	38	0.39	Yin yang huo
MOL000359	Sitosterol	36.9	0.75	Yin yang huo
MOL000422	Kaempferol	41.9	0.24	Yin yang huo
MOL004367	Olivil	62.2	0.41	Yin yang huo
MOL004373	Anhydroicaritin	45.4	0.44	Yin yang huo
MOL004380	C-Homoerythrinan,1,6-didehydro-3,15,16-trimethoxy-, (3. Beta)-	39.1	0.49	Yin yang huo
MOL004382	Yin yang huo A	57	0.77	Yin yang huo
MOL004384	Yin yang huo C	45.7	0.5	Yin yang huo
MOL004386	Yin yang huo E	51.6	0.55	Yin yang huo
MOL004388	6-Hydroxy-11,12-dimethoxy-2,2-dimethyl-1,8-dioxo-2,3,4,8-tetrahydro-1h-isochromeno[3,4-h] isoquinolin-2-ium	60.6	0.66	Yin yang huo
MOL004391	8-(3-Methylbut-2-enyl)-2-phenyl-chromone	48.5	0.25	Yin yang huo
MOL004394	Anhydroicaritin-3-O-alpha-L-rhamnoside	41.6	0.61	Yin yang huo
MOL004396	1,2-bis(4-hydroxy-3-methoxyphenyl) propan-1,3-diol	52.3	0.22	Yin yang huo
MOL004425	Icariin	41.6	0.61	Yin yang huo
MOL004427	Icariside A7	31.9	0.86	Yin yang huo
MOL000006	Luteolin	36.2	0.25	Yin yang huo
MOL000622	Magnograndiolide	63.7	0.19	Yin yang huo
MOL000098	Quercetin	46.4	0.28	Yin yang huo
MOL001677	Asperglaucide	58	0.52	Zhi mu
MOL001944	Marmesin	50.3	0.18	Zhi mu
MOL003773	Mangiferolic acid	36.2	0.84	Zhi mu
MOL000422	Kaempferol	41.9	0.24	Zhi mu
MOL004373	Anhydroicaritin	45.4	0.44	Zhi mu
MOL004489	Anemarsaponin F_qt	60.1	0.79	Zhi mu
MOL004492	Chrysanthemaxanthin	38.7	0.58	Zhi mu
MOL004497	Hippeastrine	51.7	0.62	Zhi mu
MOL004514	Timosaponin B III_qt	35.3	0.87	Zhi mu
MOL000449	Stigmasterol	43.8	0.76	Zhi mu
MOL004528	Icariin I	41.6	0.61	Zhi mu
MOL004540	Anemarsaponin C_qt	35.5	0.87	Zhi mu
MOL004542	Anemarsaponin E_qt	30.7	0.86	Zhi mu
MOL000483	(Z)-3-(4-hydroxy-3-methoxy-phenyl)-N-[2-(4-hydroxyphenyl) ethyl] acrylamide	118	0.26	Zhi mu
MOL000546	Diosgenin	80.9	0.81	Zhi mu
MOL000631	Coumaroyltyramine	113	0.2	Zhi mu
MOL007107	C09092	36.07	0.25	Dan shen
MOL007108	Isocryptotanshi-none	54.98	0.39	Dan shen
MOL007111	Isotanshinone II	49.92	0.4	Dan shen
MOL007115	Manool	45.04	0.2	Dan shen
MOL007118	Microstegiol	39.61	0.28	Dan shen
MOL007119	Miltionone I	49.68	0.32	Dan shen
MOL007120	Miltionone II	71.03	0.44	Dan shen
MOL007121	Miltipolone	36.56	0.37	Dan shen
MOL007122	Miltirone	38.76	0.25	Dan shen
MOL007123	Miltirone II	44.95	0.24	Dan shen
MOL007124	Neocryptotanshinone ii	39.46	0.23	Dan shen
MOL007125	Neocryptotanshinone	52.49	0.32	Dan shen
MOL007127	1-Methyl-8,9-dihydro-7h-naphtho[5,6-g] benzofuran-6,10,11-trione	34.72	0.37	Dan shen
MOL007130	Prolithospermic acid	64.37	0.31	Dan shen
MOL007132	(2R)-3-(3,4-dihydroxyphenyl)-2-[(Z)-3-(3,4-dihydroxyphenyl) acryloyl] oxy-propionic acid	109.38	0.35	Dan shen
MOL007140	(Z)-3-[2-[(E)-2-(3,4-dihydroxyphenyl) vinyl]-3,4-dihydroxy-phenyl] acrylic acid	88.54	0.26	Dan shen
MOL007141	Salvianolic acid g	45.56	0.61	Dan shen
MOL007142	Salvianolic acid j	43.38	0.72	Dan shen
MOL007143	Salvilenone I	32.43	0.23	Dan shen
MOL007145	Salviolone	31.72	0.24	Dan shen
MOL007149	NSC 122421	34.49	0.28	Dan shen
MOL007150	(6S)-6-hydroxy-1-methyl-6-methylol-8,9-dihydro-7h-naphtho[8,7-g] benzofuran-10,11-quinone	75.39	0.46	Dan shen
MOL007151	Tanshindiol B	42.67	0.45	Dan shen
MOL007152	Przewaquinone E	42.85	0.45	Dan shen
MOL007154	Tanshinone iia	49.89	0.4	Dan shen
MOL007155	(6S)-6-(hydroxymethyl)-1,6-dimethyl-8,9-dihydro-7h-naphtho[8,7-g] benzofuran-10,11-dione	65.26	0.45	Dan shen
MOL007156	Tanshinone VI	45.64	0.3	Dan shen
MOL002897	Epiberberine	43.09	0.78	Huang lian
MOL002903	(R)-canadine	55.37	0.77	Huang lian
MOL002904	Berlambine	36.68	0.82	Huang lian
MOL002907	Corchoroside A_qt	104.95	0.78	Huang lian
MOL000622	Magnograndiolide	63.71	0.19	Huang lian
MOL000762	Palmidin A	35.36	0.65	Huang lian
MOL000785	Palmatine	64.6	0.65	Huang lian
MOL000098	Quercetin	46.43	0.28	Huang lian
MOL001458	Coptisine	30.67	0.86	Huang lian
MOL002668	Worenine	45.83	0.87	Huang lian
MOL008647	Moupinamide	86.71	0.26	Huang lian

**Table 2:** The active component and key target of RSRN.

Compound	Type	Betweenness	Closeness	Degree	Eigenvector
Quercetin	Mol	6956.367	0.001616	426	0.172085
Kaempferol	Mol	1074.972	0.001422	114	0.093268
Stigmasterol	Mol	234.9962	0.001351	112	0.071736
Luteolin	Mol	966.566	0.001414	110	0.077567
Beta-sitosterol	Mol	188.0214	0.001361	88	0.076787
Anhydroicaritin	Mol	173.2552	0.001372	66	0.083024
Danshinone II A	Mol	1130.871	0.00122	41	0.073208
Dehydrotanshinone II A	Mol	26.81371	0.001348	40	0.06437
Palmatine	Mol	16.99011	0.001297	34	0.054885
C-homoerythrinan, 1,6-didehydro-3,15,16-trimethoxy-, (3. Beta.)-	Mol	292.7947	0.001333	32	0.070996
PTGS2	Gene	6853.524	0.001597	231	0.158477
PTGS1	Gene	1835.112	0.001504	148	0.126258
SCN5A	Gene	1382.629	0.001497	137	0.127246
HSP90AA1	Gene	2411.688	0.001497	131	0.107942
NCOA2	Gene	2395.628	0.001481	130	0.098189
ADRB2	Gene	1468.898	0.001495	127	0.12509

## Data Availability

All the data generated or analyzed during this study are included within the paper.

## Abbreviations

ADME: Absorption, distribution, metabolism, and excretion
OB: Oral bioavailability
DL: Drug-likeness
GO: Gene ontology
KEGG: Kyoto encyclopedia of genes and genomes
CC: Cellular components
MF: Molecular functions
BP: Biological processes
TCMSP: Traditional Chinese medicine systems pharmacology database and analysis platform.
